# Brown-Colored Malignant Pleural Fluid With High Bilirubin Levels: A Case Series

**DOI:** 10.1155/crpu/5807681

**Published:** 2024-11-18

**Authors:** Nai-Chien Huan, Larry Ellee Nyanti, Xin Ying Lee, Hema Yamini Ramarmuty, Daniel Theng Sheng Eng, Kunji Kannan Sivaraman Kannan, Yun Chor Gary Lee

**Affiliations:** ^1^Department of Respiratory Medicine, Queen Elizabeth Hospital, Kota Kinabalu, Sabah, Malaysia; ^2^Medical Department, University Malaysia Sabah, Kota Kinabalu, Sabah, Malaysia; ^3^Department of Medicine, Queen Elizabeth Hospital, Kota Kinabalu, Sabah, Malaysia; ^4^Department of Respiratory Medicine, Sir Charles Gairdner Hospital, Nedlands, Western Australia, Australia; ^5^Faculty of Health and Medical Sciences, University of Western Australia, Crawley, Western Australia, Australia; ^6^Pleural Medicine Unit, Institute for Respiratory Health, Perth, Western Australia, Australia

## Abstract

Brown-colored pleural effusion is rare and may result from high bilirubin levels such as bilothorax (often described as a pleural fluid-to-serum bilirubin ratio of > 1.0). We describe four patients with malignant pleural effusion that appeared macroscopically brown with a pleural fluid-to-serum bilirubin ratio between 3.7 and 16.2. All had metastatic adenocarcinomas; three were from lung and one from gastric origin. None demonstrated clear pleurobiliary fistulas on investigations. Postulates for the development of brown effusion include heme oxygenase 1 overexpression in malignant cells situated in the pleura, intrapleural hemolysis, passive movement of bile through microscopic diaphragmatic pores, and drainage of biliary fluid into the pleural lymphatics.

## 1. Introduction

Brown-colored pleural effusion is uncommon. A high bilirubin content is one of the possible causes. An underlying bile leak into the pleural space, known as bilothorax (or cholethorax, thoracobilia, and pleurobilia), is one of the possible etiologies of high pleural fluid bilirubin levels [1]. A raised pleural fluid-to-serum bilirubin ratio of > 1.0 is often used to define bilothorax [1]. Causes of bilothorax include biliary tract injury resulting in pleurobiliary fistula or from a subphrenic abscess formation. Brown pleural effusion has not been extensively reported in the setting of adenocarcinoma. We describe four patients with brown pleural effusions with biochemically proven high bilirubin contents and metastatic pleural adenocarcinoma but with no evidence of direct bile leak into the pleural cavity.

## 2. Case 1

**Figure 1:**
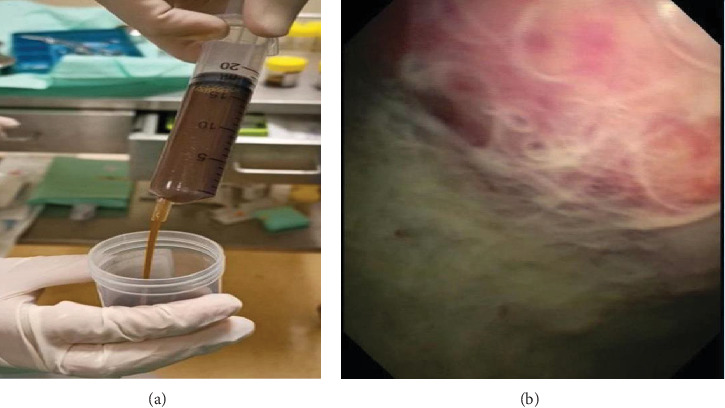
(a) Brownish pleural fluid was removed during thoracentesis. (b) Pleuroscopy showed a thickened and erythematous right parietal pleura that was covered with fibrin and brownish pleural fluid.

A 64-year-old woman presented with a chronic cough, anorexia, weight loss, and worsening dyspnea for a month. She never smoked and had no family history of malignancy. Physical examination and chest radiograph revealed a massive right pleural effusion. A computed tomography (CT) scan found no underlying lung or solid intra-abdominal lesions. Thoracentesis revealed a brown effusion (Figure 1(a)). Pleural fluid analysis was consistent with exudative (by Light's criteria) with a high pleural fluid bilirubin level of 62.7 *μ*mol/L (serum bilirubin 17.1 *μ*mol/L, ratio: 3.7; pleural fluid conjugated bilirubin 12.4 *μ*mol/L). Pleural fluid cultures and cytology were negative. A magnetic resonance cholangiopancreatography (MRCP) demonstrated no pleurobiliary fistula. Flexible bronchoscopy showed no endobronchial lesions. Pleuroscopy showed turbid brown fluid and an inflamed and erythematous right parietal pleura which was covered with fibrin (Figure 1(b)). Pleural biopsies revealed malignant cells that stained positive for metastatic lung adenocarcinoma. She continued her chemotherapy treatment and remained well with no fluid recurrence after 6 months of follow-up.

## 3. Case 2

**Figure 2:**
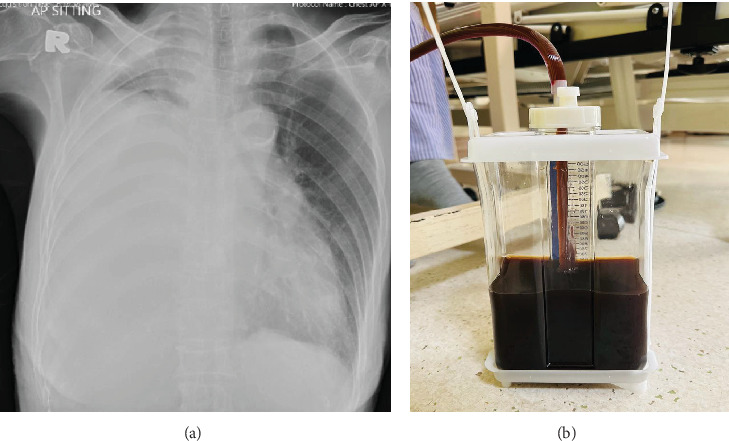
(a) Chest radiograph on arrival showed a right pleural effusion. (b) Dark brownish fluid was drained from the right pleural cavity.

A 77-year-old woman with known metastatic lung adenocarcinoma presented to the emergency department with a 2-week history of dyspnea. The chest radiograph showed a right pleural effusion (Figure 2(a)). Thoracentesis and subsequently chest tube thoracostomy were performed. The color of the drained pleural fluid was dark brown (Figure 2(b)). Pleural fluid studies showed an exudate with a bilirubin level of 133.8 *μ*mol/L (serum bilirubin 11.2 *μ*mol/L, ratio: 11.9). CT thorax and abdomen were initially suspicious of a periampullary tumor. MRCP performed subsequently however showed no pleurobiliary fistula, and the enhancing lesion at the periampullary region may represent thickened duodenal mucosa. Duodenoscopy and endoscopic ultrasound showed no evidence of periampullary mass or biliary obstruction. She was subsequently discharged and remained well with no fluid recurrence at follow-up after 4 months and continued her oncology treatment.

## 4. Case 3

**Figure 3:**
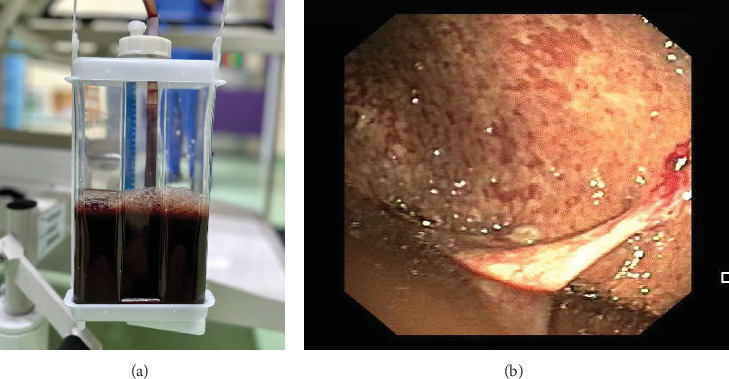
(a) Dark brownish pleural fluid was removed from the patient's left pleural cavity. (b) Pleuroscopy demonstrated a diffusely thickened left parietal pleura lined with multiple whitish and erythematous patches that were hard in consistency.

A 54-year-old gentleman presented with a 2-week history of worsening dyspnea. He had gastric pylorus carcinoma resected 3 years ago and had no local or distant recurrence on the most recent CT thorax and abdomen performed 6 months ago. Bedside ultrasonography revealed only minimal left pleural fluid. He was treated empirically for a parapneumonic pleural effusion with intravenous antibiotics before discharge. However, 3 weeks later, he developed a massive left pleural effusion necessitating chest tube thoracostomy which revealed brown-colored pleural effusion with a bilirubin level of 140 *μ*mol/L (serum total bilirubin 8.6 *μ*mol/L, ratio: 16.2) (Figure 3(a)). Pleural fluid cultures and cytology were negative. Pleuroscopy showed a diffusely thickened parietal pleura, and biopsies revealed pleomorphic neoplastic cells in loose clusters or pseudopapilla patterns (Figure 3(b)). The morphology and immunohistochemistry were similar to his primary gastric cancer. He deteriorated and died from his illness after 2 weeks of hospitalization.

## 5. Case 4

**Figure 4:**
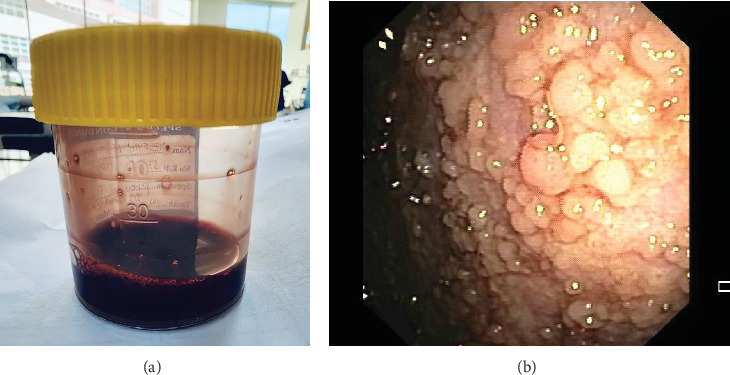
(a) Dark brownish pleural fluid was drained from the left pleural cavity. (b) Pleuroscopy demonstrated a thickened left parietal pleura lined with multiple irregular nodules.

A 68-year-old woman presented with a 3-month history of cough, 10-kg weight loss, anorexia, and worsening dyspnea. Her chest radiograph showed a massive left pleural effusion. The pleural fluid appeared macroscopically brown with a pleural fluid bilirubin level of 94.6 *μ*mol/L (serum total bilirubin 8.0 *μ*mol/L, ratio: 11.8; pleural fluid conjugated bilirubin 42.4 *μ*mol/L) (Figure 4(a)). Ultrasonography of the abdomen showed no biliary tract pathology. Thickened parietal pleura with multiple irregular nodules was observed during pleuroscopy which were TTF-1 positive, consistent with metastatic lung adenocarcinoma (Figure 4(b)). Fouchet's staining was performed but did not demonstrate any endogenous bilirubin within the malignant cells. She continued her chemotherapy treatment and remained well with no fluid recurrence after 3 months.

## 6. Discussion

We report four cases of brown-colored pleural fluid associated with very high bilirubin levels in patients with metastatic pleural carcinomas. The source of bilirubin in the pleural fluid remains unknown. The very high pleural fluid-to-serum bilirubin ratios (3.7–16.2) essentially ruled out bilirubin from peripheral blood as the majority source of bilirubin in the pleural fluid. Further investigations such as hepatobiliary ultrasonography and MRCP failed to demonstrate any direct communication between the hepatobiliary system and the pleural cavity. This raised the possibility of a local source of bilirubin from within the pleural cavity and its metastatic cancer. There are no easy or widely accepted means to establish the cellular source of bilirubin.

Nevertheless, endogenous bilirubin production by cancer cells remains a possibility. Heme oxygenase (HO-1) is known to be overexpressed in some malignant cells. HO-1 is a microsomal protein that degrades heme to bilirubin [2]. As a result of the high metabolic rate of cancer cells, oxidative stress and an increase in cytokines and growth factors lead to HO-1 overexpression [3]. Fernandes et al. described a similar case of dark-colored pleural effusion (with raised pleural fluid bilirubin levels at 119 *μ*mol/L) as the presenting feature of lung adenocarcinoma [4]. The authors suggested hemolysis as the potential reason for dark fluid stains although pleural fluid hematocrit was low at 8%. Another study by Ugurman et al. demonstrated only marginal increments in pleural fluid-to-serum bilirubin ratios when whole blood was added to pleural fluid samples [5]. Intrapleural hemolysis following malignancy-related hemorrhage can contribute to raised pleural bilirubin levels but is unlikely to be the sole mechanism in our cases. Other possible routes of bile entry into the pleural cavity include passive movement of bile through the diaphragmatic pores and drainage of biliary fluid into pleural lymphatics via pleuroperitoneal lymphatic connections [1]. Kurahara and Nagayama postulated that diaphragmatic defects caused by hepatic metastases were responsible in their patient who developed bilothorax with underlying lung adenocarcinoma [6]. This was not applicable in our reported cases.

Bilirubin has shown antioxidative and anti-inflammatory properties by inducing apoptosis and thereby inhibiting cancer cell growth [7]. Studies have verified that pancreatic [8], breast [9], and lung cancer [7] patients with raised serum bilirubin levels have longer overall survival rates than those with normal bilirubin levels. Prognostic values of pleural fluid bilirubin levels should be explored. All our patients were commenced on prophylactic intravenous antibiotics (although none developed frank pleural infections) as genuine bile leak into the pleural cavity is associated with the development of empyema. Whether patients with high bilirubin levels but without apparent fistula require antibiotics remains unknown.

## Data Availability

The data that support the findings of this study are available from the corresponding author upon reasonable request.
